# The TLR4-Active Morphine Metabolite Morphine-3-Glucuronide Does Not Elicit Macrophage Classical Activation *In Vitro*

**DOI:** 10.3389/fphar.2016.00441

**Published:** 2016-11-17

**Authors:** Samira Khabbazi, Nan Xie, Wenjun Pu, Yannick Goumon, Marie-Odile Parat

**Affiliations:** ^1^Pharmacy Australia Centre of Excellence, School of Pharmacy, University of Queensland, WoolloongabbaQLD, Australia; ^2^CNRS UPR3212, Institut des Neurosciences Cellulaires et Intégratives, Centre National de la Recherche Scientifique–University of StrasbourgStrasbourg, France

**Keywords:** classically activated macrophages, tumor-associated macrophages, morphine-3-glucuronide, toll like receptor 4, lipopolysaccharide, interferon gamma, THP1, RAW267.4

## Abstract

Macrophages are abundant in the tumor microenvironment where they adopt a pro-tumor phenotype following alternative polarization induced by paracrine factors from cancer and stromal cells. In contrast, classically activated macrophages have tumoricidal activities, such that the polarization of tumor-associated macrophages has become a novel therapeutic target. Toll-like receptor 4 engagement promotes classical activation of macrophages, and recent literature suggests TLR4 agonism to prevent metastasis and promote survival in experimental metastasis models. A growing number of studies indicate that TLR4 can respond to opioids, including the opioid receptor-inactive morphine metabolite morphine-3-glucuronide (M3G). We measured the activation of TLR4 in a reporter cell line exogenously expressing TLR4 and TLR4 co-receptors, and confirmed that M3G weakly but significantly activates TLR4. We hypothesized that M3G would promote the expression of classical activation signature genes in macrophages *in vitro*. We exposed mouse and human macrophage cell lines to M3G or the TLR4 activator lipopolysaccharide (LPS), alone or in combination with interferon gamma (IFN-γ). The classical macrophage activation markers tested were iNOS, CD86, IL-6, or TNF-α in RAW 264.7 cells and IL-6, IL-12, IL-23, TNF-α, CXCL10, and CXCL11 in THP1 cells. Our results show that despite exhibiting TLR4-activation ability, M3G does not elicit the expression of classical activation markers in LPS-responsive macrophages.

## Introduction

The possibility that the pain management of cancer surgery patients can be exploited to significantly reduce the risk of local or metastatic recurrence is generating tremendous interest. Opioids still play a major role in perioperative patient care, however, they are reported to modulate tumor growth and metastasis, with discrepant evidence from both *in vitro* and *in vivo* experimental settings (Afsharimani et al., 2011, 2015). Morphine has been tested for its effect on cancer cells, immune cells and endothelial cells grown individually (reviewed in Afsharimani et al., 2011; Xie and Parat, 2015). Furthermore, we have unveiled the ability of morphine to modulate the interaction between cancer and non-cancer cells important in the tumor microenvironment, including endothelial cells and macrophages (Afsharimani et al., 2014; Khabbazi et al., 2015, 2016).

Tumor-associated macrophages (TAMs) are critical determinants of cancer cell invasiveness, their metastatic potential as well as angiogenesis (Noy and Pollard, 2014; Bronte and Murray, 2015). Macrophages display great functional plasticity in response to specific pathological contexts (Murray et al., 2014) and play a key role in the biology of solid tumors where they constitute up to 50% of the cell population (Solinas et al., 2009). Alternatively activated (M2) macrophages promote tumor aggressiveness. In contrast, classically activated (M1) macrophages are considered to have anti-tumor properties (Noy and Pollard, 2014). Reprogramming TAMs from an M2-like phenotype to an M1-like, pro-inflammatory phenotype has the potential to induce anti-tumor activity by rendering the tumor immunogenic (Georgoudaki et al., 2016).

TLR4 plays a key role in macrophage M1 polarization. TLR4 responds to gram negative bacteria membrane component lipopolysaccharide (LPS), which binds to co-receptor MD2. After activation at the cell surface by LPS, TRL4 activates two signaling pathways. The first one, *via* the adaptor proteins Toll-IL-1 receptor domain-containing adaptor protein (TIRAP) and myeloid differentiation factor 88 (MyD88) results in the induction of pro-inflammatory cytokines. The second one *via* TRIF-related adaptor molecule (TRAM) and Toll/IL-1 receptor containing adaptor inducing IFN-β (TRIF) mediates the induction of type I interferons (O’Neill et al., 2013). LPS elicits classical activation gene expression alone or synergistically with IFN-γ. TLR4 activation by other ligands, such as the DAMP HMG1 protein also reprograms macrophages toward a M1 phenotype (Su et al., 2016).

We have previously demonstrated the ability of morphine to prevent macrophage M2 polarization elicited by tumor cells (Khabbazi et al., 2015). In the current study, we hypothesized that the opioid receptor-inactive morphine metabolite morphine-3-glucuronide (M3G), which is documented to be active at TLR4 (Hutchinson et al., 2010; Stevens et al., 2013), would modulate the M1 polarization of macrophages *in vitro*.

## Materials and Methods

### Materials

Cell culture medium, serum and supplements, primers and real-time PCR reagents were from Life Technologies (Mulgrave, VIC, Australia). M3G was from Novachem (Collingwood, VIC, Australia). QUANTI-Blue^TM^, Normocin^TM^, and HEK-Blue^TM^ Selection reagent were from Jomar Life Research (Scoresby, VIC, Australia). Other reagents were purchased from Sigma-Aldrich (Castle Hill, NSW, Australia) unless otherwise specified.

### Cell Culture

The TLR4 reporter cell line HEK-Blue^TM^-hTLR4 (Jomar Life Research, Scoresby, VIC, Australia) were cultured in Dulbecco’s modified Eagle’s medium (DMEM) supplemented with 10% (v/v) FBS, 50 U/ml penicillin, 50 μg/ml streptomycin, 100 μg/ml Normocin^TM^ and 1X HEK-Blue^TM^ Selection reagent. These human embryonic kidney 293 cells are engineered to express hTLR4 and MD-2/CD14 co-receptor genes, together with a SEAP reporter gene under NF-κB and AP-1 control. THP1 human monocytes were grown in Roswell Park Memorial Institute medium (RPMI-1640) medium supplemented with 10% FBS (v/v), penicillin (100 units/ml) and streptomycin (100 μg/ml). In some experiments, THP1 differentiation was induced by PMA as follows: THP1 cells were treated with 50 nM PMA in RPMI-1640 containing 10% (v/v) FBS, 50 units/ml penicillin, 50 μg/ml streptomycin for 48 h. The medium was replaced with serum-free RPMI medium, and cells were incubated with different concentrations of M3G (1, 5, 10, or 20 μM) or LPS (0.001–10 ng/ml) alone or in combination with 10 ng/ml IFN-γ for another 12 h. Mouse RAW264.7 macrophages were seeded in DMEM containing 5% FBS (v/v), penicillin (100 units/ml) and streptomycin (100 μg/ml) and incubated overnight. Cells were further treated with different concentrations of M3G (1, 5, 10, or 20 μM) or 0.01 ng/ml LPS alone or in combination with 1 ng/ml IFN-γ in serum-free DMEM medium for 12 h. All cell lines were kept in an incubator with a humidified atmosphere (37°C) and 5% carbon dioxide (CO_2_).

### HEK-Blue^TM^-hTLR4 Assay (TLR4 Signaling Assay)

The determination of TLR4 activation in HEK-Blue^TM^-hTLR4 cells relies on NF-κB-dependent production of SEAP detected using a colorimetric substrate placed in the cell culture medium. The cells were seeded (10,000 cells/well) in a 96-well plate and incubated for 48 h. The medium was replaced with serum-free medium containing the drugs to be tested, and cells incubated for 12 h. The supernatant of treated cells (20 μl/well) was added to QUANTI-Blue^TM^ substrate pre-warmed at 37°C (180 μl/well) and incubated for 4 h at 37°C. The plates were read at a wavelength of 655 nm with a spectrophotometer (Bio-Rad Laboratories Inc.). Results are expressed as the percentage of absorbance at 655 nm observed for control cells.

### Quantitative RT-PCR

In order to detect and quantify the expression of transcripts of specific genes, real-time reverse transcriptase polymerase chain reaction (Real time RT-PCR) was performed. Total RNA was isolated from RAW264.7 or THP1 cells and purified using a Pure link^TM^ RNA Mini Kit (Life Technologies, Melbourne, VIC, Australia). Purified RNA concentration then was determined via 260 nm absorbance (A260) using a nano-drop spectrophotometer (Thermo Fisher Scientific, Scoresby, VIC, Australia). Isolated RNA (2000 ng) was reverse transcribed to complementary DNA using High-Capacity cDNA reverse transcription kit (Life Technologies, Melbourne, VIC, Australia). Amplification and quantification of cDNA was assessed, using TaqMan^TM^ Fast Universal PCR Master Mix (Life Technologies, VIC, Australia) with AmpliTaq Gold^TM^ DNA Polymerase and TaqMan^TM^ Gene Expression Assays including human primers : IL-6 (Hs00985639-m1), IL-12 (Hs01011518-m1), IL-23 (Hs00900828-g1), TNFα (Hs01113624-g1), CXCL10 (Hs01124252-g1), and CXCL11(Hs04187682-g1) or mouse primers: iNOS (Mm00440502-m1), IL-6 (Mm00446190-m1), TNFα (Mm00443258-m1), CD86 (Mm00444543-m1) in a StepOnePlus 7500 real time PCR system (Applied Biosystems, Carlsbad, CA, USA). Quantification was performed using the comparative critical threshold (Ct) method in which the amount of target gene is normalized to 18S ribosomal RNA (18S rRNA) control gene (Schmittgen and Livak, 2008).

### Statistical Analysis

Data are shown as mean ± SEM of data obtained in at least 3 independent experiments unless otherwise indicated in the figure legend. Statistical analysis was performed on all data using Graphpad Prism software (version 6.04). Differences between group means were compared using one-way ANOVA or one-tailed Student’s *t*-test where appropriate. Differences were considered statistically significant when *p* < 0.05.

## Results

### M3G Activates TLR4, but Inhibits LPS-Induced TLR4 Activation in a Reporter Cell Line

We made use of the HEK-Blue^TM^ cells to measure TLR4 activation (**Supplementary Figure 1**). A dose–response curve of LPS was first generated (**Supplementary Figure 1A**) and showed a maximal activation of ∼500% compared to untreated cells. We tested the ability of M3G to activate TLR4 in this system (**Supplementary Figure 1B**) and showed at 1 μM and above a modest but statistically significant activation plateauing at ∼150–160% of control with maximal activation at and above 10 μM. We verified that apparent TLR4 activation was not due to LPS contamination of the M3G solution. To that extent, we repeated the activation by M3G in the presence of the LPS-binding antibiotic polymyxin B. Polymyxin B blunted the activation induced by LPS (**Supplementary Figure 1C**) but had no effect on that induced by M3G (**Supplementary Figure 1D**), confirming M3G was free from LPS. Opioid receptor agonists and antagonists have been shown to act as inhibitors of TLR4 activation by its ligand LPS (Stevens et al., 2013). We tested the ability of M3G to modulate LPS-induced TLR4 activation in this system (**Supplementary Figure 1E**). The results show significant inhibition of the activation induced by 1 ng/ml LPS by concentrations of M3G as low as 0.1 μM.

### M3G Does Not Elicit M1 Differentiation of RAW264.7 Macrophages

**FIGURE 1:**
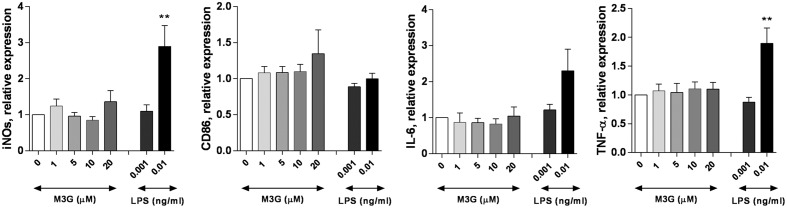
**Effect of M3G on expression of M1 markers in RAW264.7 cells.** RAW264.7 cells (2 × 10^5^ cells/well) were exposed to 0.001 or 0.01 ng/ml LPS, or M3G at the indicated concentrations (1, 5, 10, and 20 μM). The mRNA levels of iNOS, CD86, IL-6, or TNF-α were determined by qRT-PCR. Results are shown relative to control (untreated) RAW264.7 cells. Results are shown as mean ± SEM, *n* = 4–7 independent experiments. ^∗∗^*p* < 0.01, LPS vs. control cells.

**FIGURE 2:**
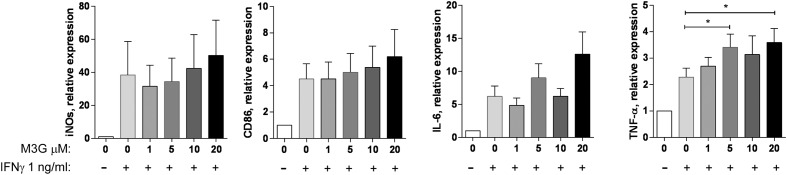
**Effect of M3G co-administered with IFN-γ on expression of M1 markers in RAW264.7 cells.** RAW264.7 cells (2 × 10^5^ cells/well) were treated with 1 ng/ml IFN-γ alone or together with M3G (1, 5, 10, or 20 μM) for 12 h. Expression of iNOS, TNF-α, IL-6, and CD86 mRNA was determined by qRT-PCR. Results are shown relative to control (untreated) RAW264.7 cells. Mean ± SEM is shown, *n* = 4 independent experiments. ^∗^*p* < 0.05, M3G + IFN-γ vs. IFN-γ alone, one-way ANOVA analysis with Dunnett’s multiple comparisons. Results are shown as mean ± SEM, *n* = 4–7 independent experiments.

To examine the effect of M3G on RAW264.7 macrophages, we first tested mRNA expression of a series of recognized M1 markers in response to LPS, interferon gamma (IFN-γ), or LPS and IFN-γ in combination (**Supplementary Figure 2**). Our aim was to use a concentration of LPS that would induce M1 differentiation, elicit a level of TLR4 activation in the range of that induced by M3G, and allow an increased effect of the combination IFN-γ + LPS compared to LPS or IFN-γ alone. The expression of inducible nitric oxide synthase (iNOS), interleukin-6 (IL-6) and tumor necrosis factor-α (TNF-α) was induced by 0.01 ng/ml LPS and the induction was enhanced by the co-administration of IFN-γ to the cells. The expression of CD86 was not increased by 0.01 ng/ml LPS but the combination LPS + IFN-γ resulted in increased expression compared to either cytokine alone. We then used these markers to evaluate the effect of M3G on M1 macrophage polarization (**Figure 1**). The results show that M3G up to a concentration of 20 μM did not affect the expression of iNOS, CD86, IL-6, or TNF-α by RAW 264.7 cells. In contrast, except for CD86, the expression of these markers was induced by 0.01 but not 0.001 ng/ml LPS. We then tested whether M3G increased the expression of M1 markers when applied in combination with IFN-γ (**Figure 2**). There was a trend toward increased M1 marker expression with increasing concentrations of M3G in the presence of IFN-γ but except in the case of TNF-α, no statistical significance was detected (One-way ANOVA analysis with Dunnett’s multiple comparisons M3G + IFN-γ vs IFN-γ alone). The results indicate the TLR4 activation by M3G is below the threshold required to elicit M1 differentiation in RAW264.7 cells, whether applied alone or in combination with IFN-γ.

### Effect of M3G on LPS-Induced TLR4 Activation in RAW264.7 Cells

**FIGURE 3:**
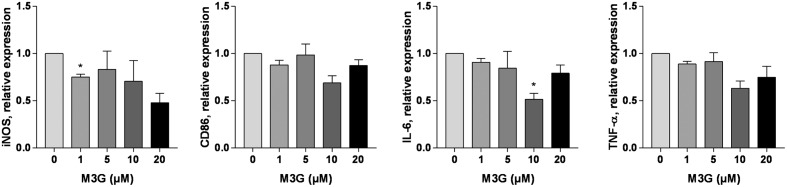
**Effect of M3G on LPS-induced TLR4 activation in RAW264.7 cells.** Cells were exposed to 1 ng/ml LPS alone or in combination with different concentrations of M3G (1, 5, 10, and 20 μM) for 12 h. The mRNA expression of M1 polarization markers was assessed by real-time RT-PCR. Results are expressed relative to LPS-treated cells. Results are shown as mean ± SEM, *n* = 3 independent experiments. ^∗^*p* < 0.05, M3G vs. control cells, One-way ANOVA analysis with Dunnett’s multiple comparisons.

Morphine-3-glucuronide acted as a weak activator of TLR4 in the reporter cell line, but also as an inhibitor of LPS-induced TLR4 activation (**Supplementary Figure 1D**). We thus tested whether M3G could alter the LPS-induced TLR4 activation in RAW264.7 cells. Cells were incubated in the presence of LPS (1 ng/ml) and M3G up to a concentration of 20 μM. The expression of iNOS, CD86, IL-6, and TNF-α was assessed (**Figure 3**). The results showed a trend toward decreased LPS induction of the tested genes by M3G. There was no statistical significance in this reduction except for iNOS with 1 μM M3G and IL-6 with 10 μM M3G (one-way ANOVA with Dunnet’s multiple comparisons, LPS + M3G vs. LPS alone). To rule out that the effect of M3G could be at TLR4 expression rather than activation level, we verified that M3G had no effect on TLR4 expression (**Supplementary Figure 3**).

### Effect of M3G on PMA-Differentiated THP1 Cells

We next tested the human cell line THP1. We first examined whether PMA differentiation of the THP1 monocytic cell line to macrophages modulated TLR4 expression and subsequently the cell response to LPS. THP1 cells were incubated in the presence or absence of 50 nM PMA for 48 h and the mRNA expression of TLR4 and a series of M1 markers was assessed using RT-PCR (**Supplementary Figure 4**). The results showed a significant increase in TLR4 expression level (∼fourfold) in PMA-treated THP1 cells compared to the control cells. Moreover, the induction of some (IL-23, IL-12, IL-6, TNF-α), but not all M1 markers tested in response to LPS was increased in PMA-differentiated THP1 cells compared to undifferentiated THP1 cells. Of note, iNOS was not induced by LPS regardless of PMA treatment, and CD86 was weakly induced. Surprisingly, CXCL10 and CXCL11 expression was induced more efficiently in undifferentiated THP1 than in PMA-differentiated THP1. We next tested the effect of LPS (0.01 ng/ml) and IFN-γ (10 ng/ml) alone or in combination on M1 marker expression in PMA-differentiated THP1 cells. The expression of IL-6, IL-12, IL-23, TNF-α, CXCL10, and CXCL11 mRNA was induced by LPS, by IFN-γ and the induction was enhanced by the co-administration of IFN-γ and LPS to the cells (**Supplementary Figure 5**).

**FIGURE 4:**
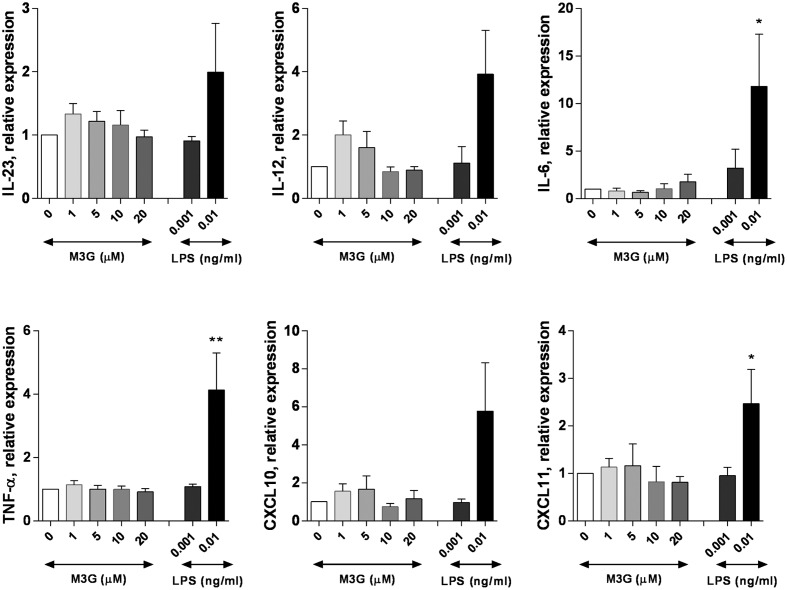
**Effect of M3G on the expression of M1 markers in PMA-differentiated THP1 cells.** THP-1 cells were incubated with 50 nM PMA for 48 h. Cells were then treated with M3G (1, 5, 10, or 20 μM) for 12 h. LPS (0.001 and 0.01 ng/ml) was also used as a comparison. The expression of IL-6, IL-12, IL-23, TNF-α, CXCL10, and CXCL11 was determined by qRT-PCR. ^∗^*p* < 0.05, ^∗∗^*p* < 0.01 LPS vs. control cells, One-way ANOVA analysis with Dunnett’s multiple comparisons. Results are shown as mean ± SEM, *n* = 4–12 independent experiments.

**FIGURE 5:**
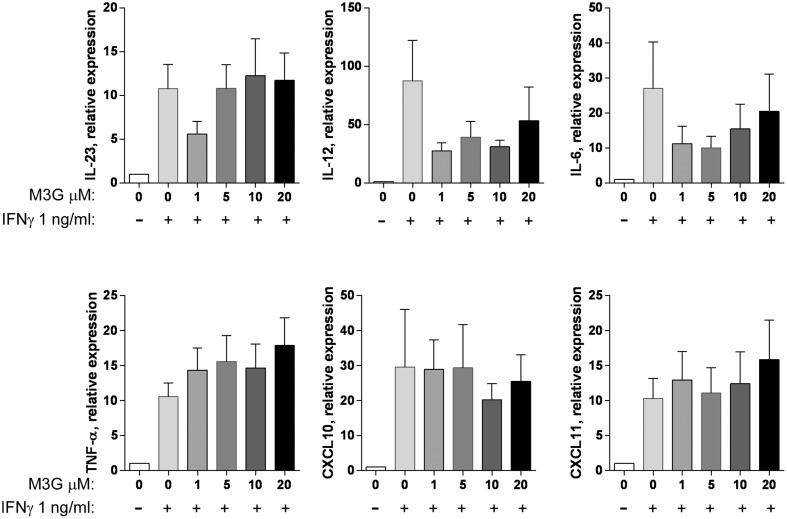
**Effect of M3G co-administered with IFN-γ on expression of M1 markers in PMA-differentiated THP1 cells.** THP-1 cells were treated with 50 nM PMA for 48 h and exposed to 10 ng/ml IFN-γ alone or in combination with different concentrations of M3G (1, 5, 10, or 20 μM) for another 12 h. The expression of IL-6, IL-12, IL-23, TNF-α, CXCL10, and CXCL11 mRNA was determined by qRT-PCR. Results are shown relative to control differentiated THP-1 cells. Results are shown as mean ± SEM, *n* = 7–11 independent experiments.

We then assessed whether M3G can induce the expression of M1 markers when applied to PMA-differentiated THP1 cells (**Figure 4**). M3G (1–20 μM) had no effect the expression of IL-6, IL-12, IL-23, TNF-α, CXCL10, or CXCL10 by THP1 cells. LPS, used as a positive control, induced increased expression of these markers at 0.01 but not 0.001 ng/ml. We then evaluated whether M3G could induce M1 polarization when co-administered with IFN-γ (**Figure 5**). None of the tested markers had increased expression in cells treated with IFN-γ + M3G up to 20 μM compared to cells treated with IFN-γ alone. These results show that M3G, whether alone or in combination with IFN-γ, does not elicit M1 polarization in PMA-treated THP1 cells.

### Effect of M3G on LPS-Induced TLR4 Activation in THP1 Cells

**FIGURE 6:**
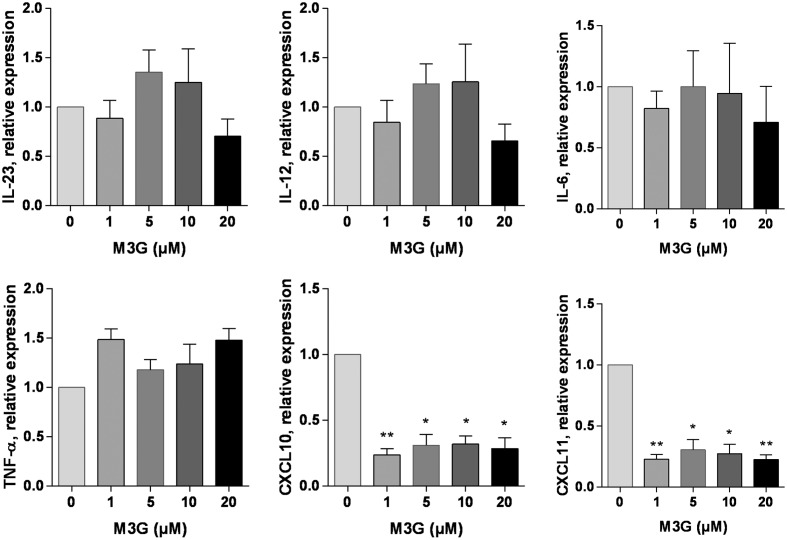
**Effect of M3G on LPS-induced TLR4 activation.** THP1 cells were treated with 50 nM PMA for 48 h and exposed to 1 ng/ml LPS alone or in combination with different concentrations of M3G (1, 5, 10, and 20 μM) for another 12 h. The mRNA expression of M1 polarization markers IL-6, IL-12, IL-23, TNF-α, CXCL10, and CXCL11 was assessed by real-time RT-PCR. Results are expressed relative to LPS-treated, PMA-differentiated THP1 cells. Results are shown as mean ± SEM, *n* = 3 independent experiments. ^∗^*p* < 0.05, ^∗∗^*p* < 0.01 M3G vs. control cells, One-way ANOVA analysis with Dunnett’s multiple comparisons.

We tested whether M3G could alter the LPS-induced TLR4 activation in PMA-differentiated THP1 cells. PMA-treated THP1 cells were incubated in the presence of LPS (1 ng/ml) and M3G up to a concentration of 20 μM. The expression of IL-6, IL-12, IL-23, TNF-α, CXCL10, and CXCL11 was assessed. There was an apparent ∼70% decrease in the LPS-induced expression of CXCL10 and CXCL11 by M3G which was statistically significant (One-way ANOVA with Dunnet’s multiple comparisons, LPS + M3G vs LPS alone). Interestingly, the effect of M3G did not seem to be dose-dependent (**Figure 6**). We verified that M3G had no effect on TLR4 expression in THP1 cells (**Supplementary Figure 3**).

### Effect of M3G on Poly ICLC-Induced M1 Polarization Marker Expression

To test whether the inhibitory effect of M3G on LPS-induced M1 marker expression was specific for the TLR4 pathway, we evaluated the effect of M3G on M1 marker induction by another TLR, namely TLR3. To that extent, we exposed RAW264.7 or THP1 to the TLR3 ligand Poly ICLC and measured mRNA expression of the markers whose expression was affected by M3G in LPS experiments, namely iNOS and IL-6 for RAW264.7 and CXCL10 and CXCL11 for THP1 cells. The results (**Supplementary Figure 6**) show no decrease of the poly ICLC-induced expression of these markers by M3G, indicating that M3G interferes somewhat selectively with TLR4-mediated, LPS-induced M1 macrophage polarization.

## Discussion

There is increasing evidence that the morphine metabolite M3G is active at TLR4 (Hutchinson et al., 2010; Lewis et al., 2010; Due et al., 2012). Long neglected by researchers because it has essentially no activity on opioid receptors and is not analgesic (Milne et al., 1996), this glucuronidated metabolite is produced in abundance in mice, peaking in the serum at ∼60 μM after i.v. or s.c. injection, and ∼100 μM after i.p. injection of 80 μmol/kg of morphine (Handal et al., 2002), and humans, ranging from 0.1 to 24 μM (Lee et al., 2015) or from 0.83 to 39 μM (Netriova et al., 2006) in morphine-treated oncological patients. Activation of TLR4 by M3G is proposed to cause neuro-inflammation and hyperalgesia via central glial and endothelial cells (Lewis et al., 2010; Grace et al., 2014). Considering the crucial role of TLR4 activation on innate immune responses, it becomes important to test whether M3G could mimic the effects of LPS on macrophage M1 polarization *in vitro*.

In our experiments, while eliciting TLR4 activation in the HEK-Blue^TM^-hTLR4 cells in a modest (at most 60% above control cells, compared to 500% of control obtained with LPS) but significant fashion, M3G strongly inhibited LPS-induced TLR4 activation in the same cell line (up to 45% inhibition at 30 and 100 μM). The activation of TLR4 is in agreement with previous findings making use of reporter lines overexpressing TLR4 (Hutchinson et al., 2010; Lewis et al., 2010). M3G activation of TLR4 has also been documented *in vitro* using cells that endogenously express TLR4 (Lewis et al., 2010; Due et al., 2012; Grace et al., 2014). The inhibition by M3G of LPS-induced activation of TLR4 is a novel finding, albeit expected since similar results have been obtained using other opioids, namely morphine, fentanyl, naltrexone, and β-funaltrexamine (β-FNA) (Stevens et al., 2013). Immunomodulating drugs targeting TLR4 are actively investigated in cancer, where TLR4 persistent activation can induce chronic inflammatory conditions contributing to carcinogenesis, while TLR4 agonists can induce anti-tumor immunity (Awasthi, 2014). A TLR4-active agent that promotes anti-tumor immunity while decreasing inflammatory response would be valuable in cancer therapy (Awasthi, 2014). It would be interesting to assess opioids in this context, including M3G, whose activity at TLR4 is proposed to be mediated by its glucuronidated moiety on the carbon number 3 (Lewis et al., 2010).

Our preliminary steps to select markers for the experiments to be conducted with RAW 264.7 and THP1 cells revealed remarkable differences in the response of these two cell lines to LPS. For example iNOS was strongly induced in the mouse but not the human cell line, an interspecies divergence that has been previously reported (Schroder et al., 2012; Spiller et al., 2016). We chose markers whose induction with 0.01 ng/ml LPS was detectable, and further increased with co-treatment by IFN-γ (**Figures 2** and **6**). In THP1, the mRNA expression of CXCL10 and CXCL11 was induced by LPS to a higher extent in undifferentiated compared to PMA-differentiated cells, even though PMA resulted in a ∼ fourfold increase in TLR4 mRNA expression. We nonetheless included CXCL10 and CXCL11 in our panel of M1 markers because they were both significantly induced by LPS in PMA-differentiated cells, and they have been reported to be the genes with the most similar pattern of expression among all source of macrophages stimulated *in vitro* with LPS and IFN-γ (Spiller et al., 2016), indicating they are a reliable experimental polarization marker.

Our results indicate that neither RAW 264.7 nor PMA-differentiated THP1 cells respond to M3G alone by increasing the expression of genes known as M1 markers. This is despite the activation seen with M3G in the reporter cell line overexpressing TLR4 and co-receptors, and despite choosing M1 markers that are induced by LPS in RAW 264.7 and PMA-differentiated THP1 in the same experimental conditions.

RAW 264.7 cells are hypo-responsive to LPS due to a point mutation affecting surface expression of the TLR4 in BALB/c mice and cells of BALB/c origin (Tsukamoto et al., 2013). Nonetheless, they are widely employed in experiments testing the effect of TLR4 activation in response to LPS and we established their responsiveness to 0.01 mg/ml LPS. Similarly negative results were obtained when RAW 264.7 cells were exposed to M3G in combination with IFN-γ, except for TNFα mRNA that was statistically higher at two concentrations of M3G + IFN-γ compared to IFN-γ alone. Likewise, THP1 cells which are extensively used to study TLR4 response *in vitro*, failed to respond to M3G alone or in combination with IFN-γ by increased expression of M1 polarization markers. These results could reflect a quantitatively and/or qualitatively different response of the TLR4 to M3G compared to LPS. Of note, we employed LPS as a control at two concentrations, one of which activated HEK-Blue^TM^-hTLR4 cells to a similar extent as M3G (0.001 ng/ml) while the other one elicited much stronger activation of the HEK-Blue^TM^-hTLR4 cells than M3G (0.01 ng/ml). The fact that LPS was able to induce M1 polarization markers at 0.01 ng/ml but not at 0.001 ng/ml, suggests that TLR4 activation by M3G is below the threshold needed for the activation of these macrophage cell lines.

*In silico* studies using the structure of the TLR4 and MD-2 complex documented that opioids dock in the LPS-binding cleft of MD-2 (Hutchinson et al., 2010). This was confirmed for M3G, with the additional information that the glucuronic acid portion of this metabolite interacts closely with residues in MD-2 known to be important for the activation of TLR4 by LPS (Lewis et al., 2010). The HEK-Blue^TM^-hTLR4 reporter cell line is co-transfected with MD2 and CD14 (in addition to TLR4) while the RAW 264.7 and THP1 cells rely on endogenously produced MD2 and CD14. This may lead to better activation of the HEK-Blue^TM^-hTLR4 than RAW 264.7 and THP1 by ligands. However, M3G has been shown to activate endogenously expressed TLR4, e.g., in the mouse BV2 glial cell line where M3G elicited increased IL1 production (Lewis et al., 2010), in central nervous system endothelial cells where it induced TNF-α, COX2, and PGE2 production (Grace et al., 2014), or in primary dorsal root ganglion neurons where increased excitability by M3G was prevented by an inhibitor of the TLR4-MD2 complex (Due et al., 2012). The possibility that primary cells such as bone marrow derived macrophages or thioglycollate medium-elicited macrophages may show a higher response to M3G than cell lines could be tested in the future to help clarify this point.

Expression or upregulation of TLR4 has been identified in tumor cells, and although it is not always the case (Lamrani et al., 2016), TLR4 activation is mostly reported to stimulate cancer cell aggressive behavior (Molteni et al., 2006; Ikebe et al., 2009; Liao et al., 2012; Chung and Kim, 2016; Sun et al., 2016). It has been proposed that activation of TLR4 on immune cell is protective, while activation of cancer cell TLR4 promotes aggressiveness (Afsharimoghaddam et al., 2016). In this context, it would be interesting to assess whether M3G increases invasiveness of cancer cell lines that express TLR4.

Inhibition of TLR4 LPS activation by opioids, both OR agonists and antagonists, has been documented. It was concluded that opioids inhibit LPS signaling in a non-competitive fashion (Stevens et al., 2013). Although M3G was not one of the opioids assessed in this study, the opioid agents tested exhibited an inhibition of LPS-induced signaling that was not concentration-dependent, and attributed to either a low capacity site easily saturated, or a type of non-competitive antagonism (Stevens et al., 2013). Our results indicate that the LPS-induced expression of IL6 and chemokines CXCL10 and CXCL11 is affected by M3G and interestingly, the inhibition does not seem to depend on the concentration of M3G. In agreement with our results, opioid receptor-independent inhibition of LPS-induced CXCL10 expression in the brain of mice by treatment with the opioid antagonist β-funaltrexamine has been documented (Davis et al., 2015).

Polarization of TAMs to the M1 phenotype can be promoted by the activation of TLR3 (Liu et al., 2016). There is currently no information on the effect of M3G on other members of the TLR family than TLR4. This gap in the literature suggests future experiments testing if M3G can activate TLR3. Our current data suggest that M3G does not prevent TLR3-mediated induction of the expression of M1 polarization markers.

Previous results from our laboratory have shown that morphine prevents macrophage M2 polarization in *in vitro* models mimicking the tumor micro-environment (Khabbazi et al., 2015). We speculated that M3G, a metabolite produced in elevated concentrations following morphine administration, would further tip the balance of tumor macrophage polarization toward the M1 end of the phenotype spectrum via its reported ability to activate the TLR4. The results of our study do not point in this direction, and the effects of M3G on innate immunity will need further evaluation, including *in vivo* experiments.

## Author Contributions

SK performed experiments and participated in their design and in manuscript writing. NX and WP performed experiments and critically edited the manuscript. YG contributed to the conception of the work and critically edited the manuscript. M-OP designed and interpreted experiments, and wrote the manuscript. All authors approved the final version and agreed to be accountable for all aspects of the work.

## Conflict of Interest Statement

The authors declare that the research was conducted in the absence of any commercial or financial relationships that could be construed as a potential conflict of interest.

## Abbreviations

DAMP: danger associated molecular pattern
HMG1: high mobility group box 1
SEAP: secreted embryonic alkaline phosphatase
