# Supplementary material for: The TLR4-Active Morphine Metabolite Morphine-3-Glucuronide Does Not Elicit Macrophage Classical Activation In Vitro
Source: Front Pharmacol. 2016 Nov 17;7:441. doi: 10.3389/fphar.2016.00441 (PMC5112272; doi:10.3389/fphar.2016.00441)
Supplement: Supplementary file 1 [file Image_1.PDF]

Supplementary Figure 1

A B

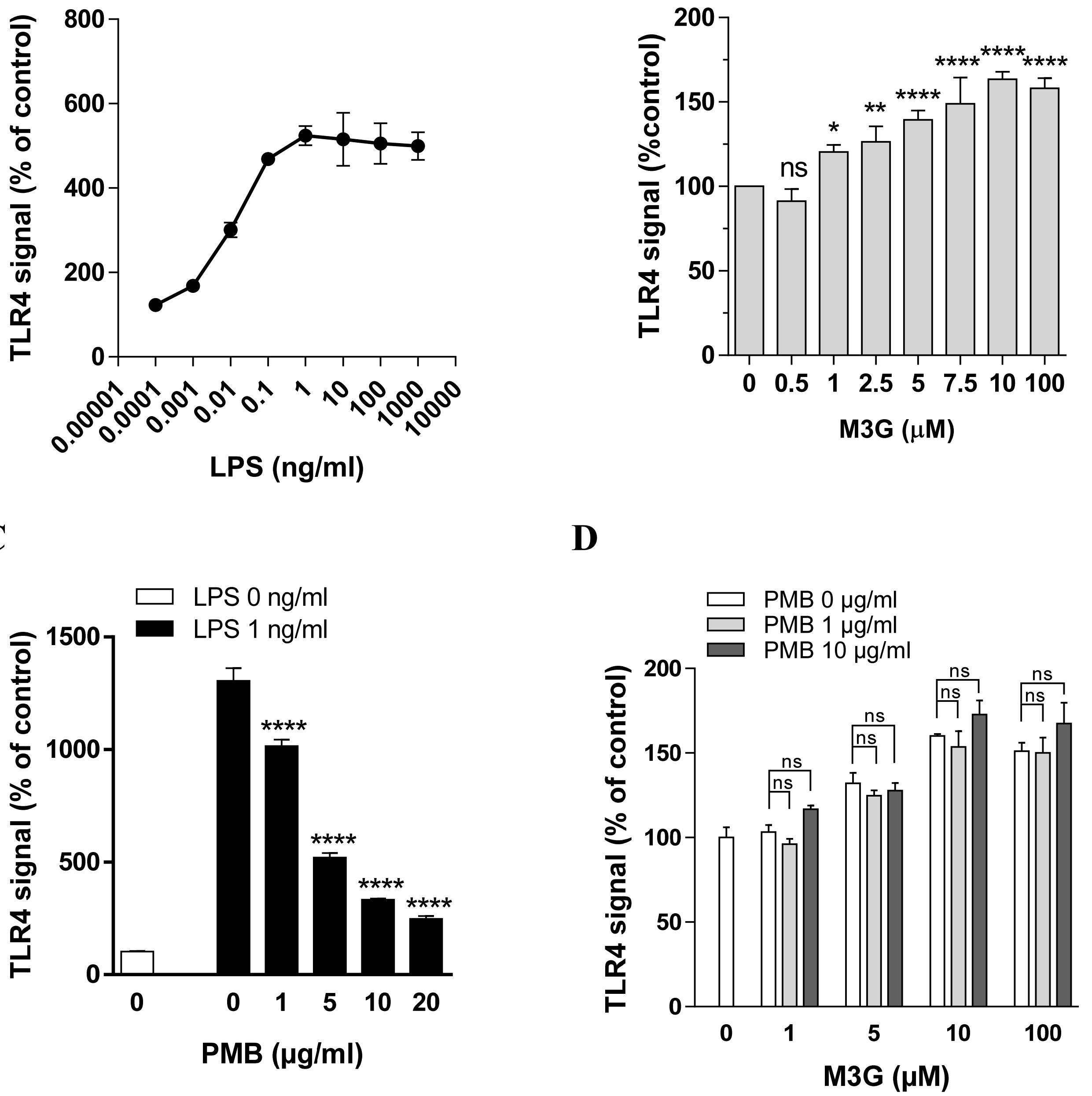

E

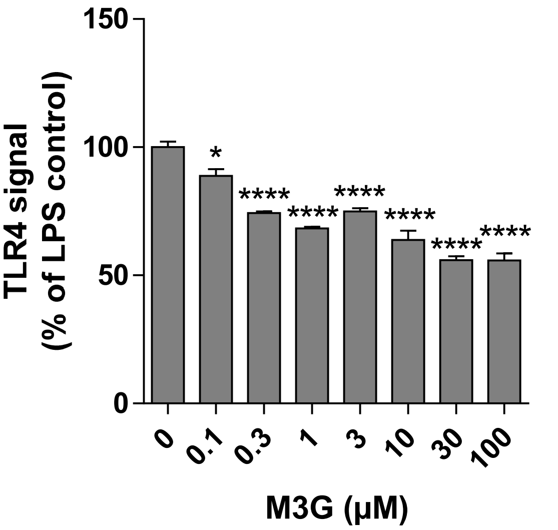

Supplementary figure 1: Effect of morphine or M3G on TLR4 activity. A) HEK-Blue™ hTLR4 cells were treated with LPS (0 to 1000 ng/ml). After 12 h incubation, 20 µl cell supernatant was added to 180 µL QUANTI-Blue™ substrate and incubated for 4 h. The TLR4 response was calculated by dividing the OD at 655 nm for the LPS treated cells by the OD at 655 nm for the LPS untreated cells (0 ng/ml, 100% TLR4 signal). B) HEK-Blue™ hTLR4 cells were stimulated with indicated concentrations of M3G for 12 h. The levels of SEAP were measured by two-step QUANTI-Blue™. *, $p<0.05$, **, $p<0.01$, ****, $p<0.0001$. M3G treatment vs control medium. Results are shown as mean ± SEM, n= 3 replicates. C) LPS can be neutralized by polymyxin B (PMB). Cells were exposed to 1 ng/ml LPS together with indicated

concentrations of PMB for 12 h. Levels of SEAP were measured and expressed as % of control, untreated cells. ****, $p<0.0001$, LPS + PMB vs LPS alone. Results are shown as mean ± SEM, n= 3 replicates. D) The effect of M3G is not altered by polymyxin B. Cells were exposed to M3G in the presence or absence of PMB at the indicated concentrations for 12 h. Levels of SEAP were measured and expressed as % of control, untreated cells. ns, no statistical significance. Results are shown as mean ± SEM, n= 3 replicates. E) HEK-Blue™ hTLR4 cells were treated with M3G in combination with 1 ng/ml LPS. After 12 h incubation, the activity of SEAP was determined by two-step QUANTI-Blue™. *, $p<0.05$, ****, $p<0.0001$, LPS + M3G vs LPS alone. Results are shown as mean ± SEM, n=3 replicates.
