# Supplementary material for: The TLR4-Active Morphine Metabolite Morphine-3-Glucuronide Does Not Elicit Macrophage Classical Activation In Vitro
Source: Front Pharmacol. 2016 Nov 17;7:441. doi: 10.3389/fphar.2016.00441 (PMC5112272; doi:10.3389/fphar.2016.00441)
Supplement: Supplementary file 2 [file Image_2.PDF]

Supplementary Figure 2

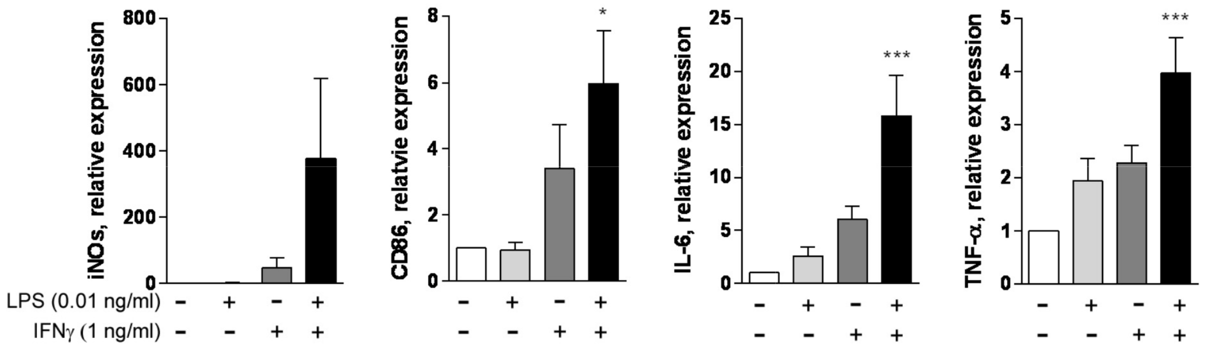

Supplementary figure 2: Induction of M1 polarization markers by LPS and IFN-γ combination in RAW264.7 cells. RAW264.7 cells seeded at 2×10^{5} cells / well were treated after 24 h with 0.01 ng/ml LPS, and/ or 1 ng/ml IFN-γ for a further 12 h. IL-6, TNF-α, CD86 and iNOS expression in response to LPS, IFN-γ alone or in combination was determined by qRT- PCR. Results are shown relative to control (untreated) RAW264.7 cells. Results are shown as mean ± SEM, n=3-5 independent experiments.*, $p<0.05$, ***, $p<0.001$, treated cells vs control cells, One way ANOVA analysis with Dunnet's multiple comparisons.
