# Supplementary material for: The TLR4-Active Morphine Metabolite Morphine-3-Glucuronide Does Not Elicit Macrophage Classical Activation In Vitro
Source: Front Pharmacol. 2016 Nov 17;7:441. doi: 10.3389/fphar.2016.00441 (PMC5112272; doi:10.3389/fphar.2016.00441)
Supplement: Supplementary file 3 [file Image_3.PDF]

Supplementary Figure 3

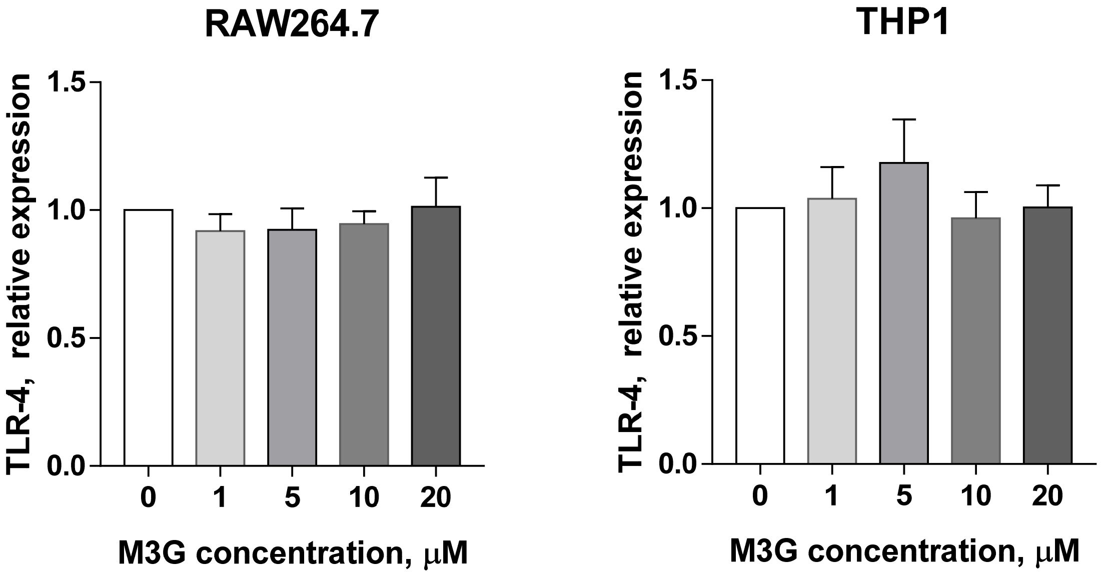

Supplementary figure 3: M3G does not modulate TLR4 expression in RAW264.7 or THP1 cells. RAW264.7 cells or PMA-differentiated THP1 cells were treated with the indicated concentrations of M3G for 12h and the expression of TLR4 was determined by qRT- PCR. Results are shown relative to control (untreated) cells. Results are shown as mean ± SEM, n=3 (THP1) or n=4 (RAW264.7) independent experiments.
