# Supplementary material for: The TLR4-Active Morphine Metabolite Morphine-3-Glucuronide Does Not Elicit Macrophage Classical Activation In Vitro
Source: Front Pharmacol. 2016 Nov 17;7:441. doi: 10.3389/fphar.2016.00441 (PMC5112272; doi:10.3389/fphar.2016.00441)
Supplement: Supplementary file 4 [file Image_4.PDF]

Supplementary Figure 4

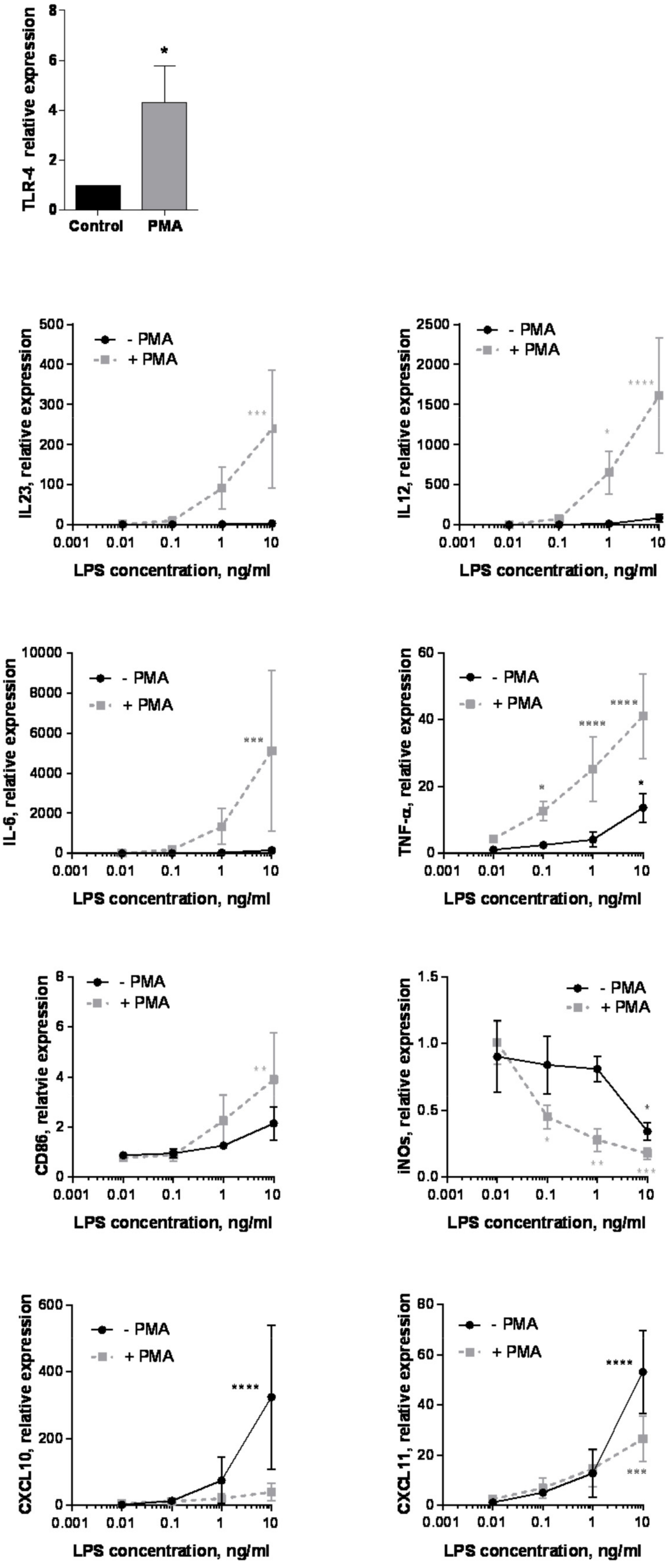

Supplementary figure 4: PMA-treated THP1 cells have increased TLR4 expression and respond to LPS. THP1 cells either remained undifferentiated or were differentiated into macrophages by treatment with 50 nM PMA for 48h. **A)** TLR4 expression was determined by qRT-PCR. Mean ± SEM is shown, n =3 independent experiments. *, $p<0.05$, one-tailed Student's t test. **B)** Undifferentiated (-PMA) or differentiated (+PMA) THP1 cells were treated with LPS (0.01-10) ng/ml for a further 12h. Expression of IL-6**,** IL-12, IL-23, iNOS, CD86, TNF-α, CXCL10 and CXCL11 mRNA was assessed by qRT-PCR. Mean ± SEM is shown, n =5-10 independent experiments. *, $p<0.05$, **, $p<0.01$, ***, $p<0.001$, ****, $p<0.0001$, LPS vs control cells, Two Way ANOVA analysis with Dunnett's multiple comparisons.
