# Supplementary material for: The TLR4-Active Morphine Metabolite Morphine-3-Glucuronide Does Not Elicit Macrophage Classical Activation In Vitro
Source: Front Pharmacol. 2016 Nov 17;7:441. doi: 10.3389/fphar.2016.00441 (PMC5112272; doi:10.3389/fphar.2016.00441)
Supplement: Supplementary file 5 [file Image_5.PDF]

Supplementary Figure 5

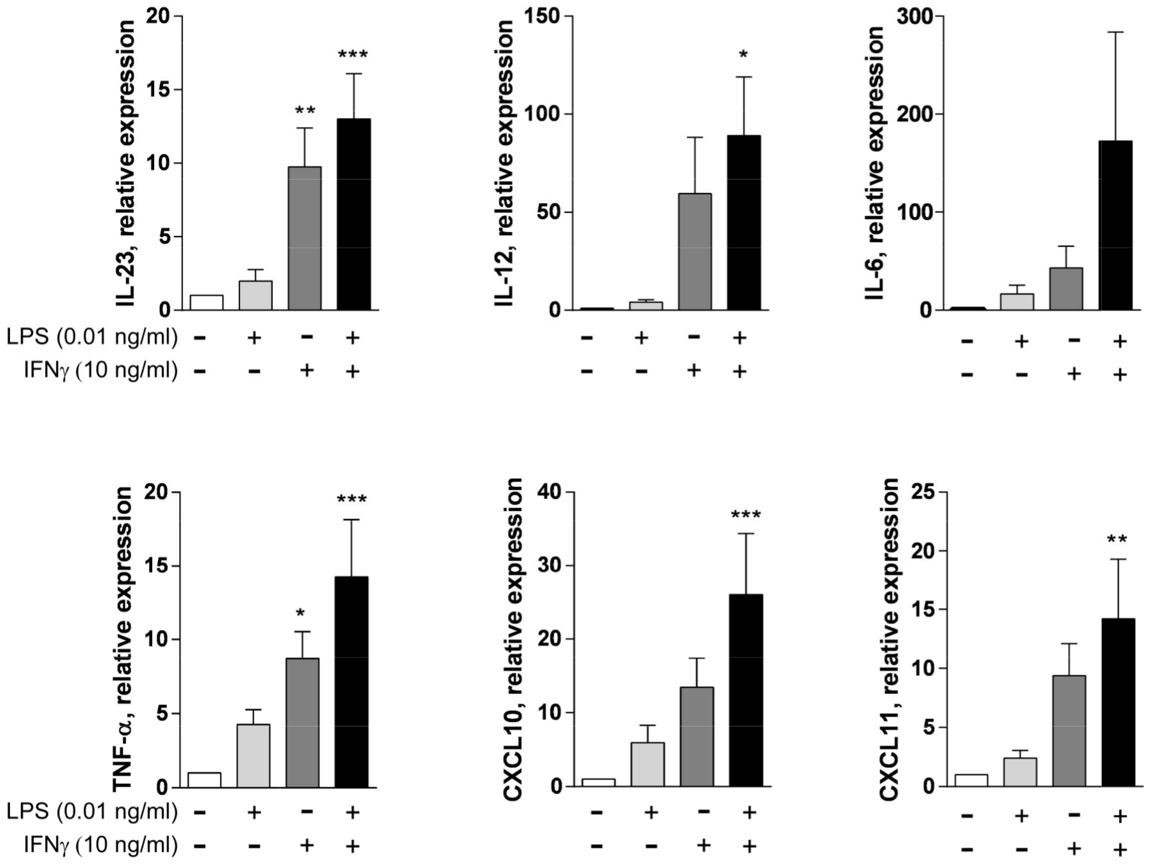

Supplementary figure 5: Expression of M1 markers in PMA-differentiated THP1 cells treated with LPS and IFN-γ alone or in combination. THP-1 cells were incubated with 50 nM PMA for 48h and then exposed to 0.01 ng/ml LPS, and/ or 1 ng/ml IFN-γ for 12h. The mRNA expression of IL-12, IL-23, IL-6, TNF-α, CXCL10 and CXCL11 was determined by qRT-PCR. Results are shown relative to control, PMA-differentiated THP-1 cells. Results are shown as mean ± SEM, n=3 independent experiments. *, $p<0.05$, **, $p<0.01$, ***,$p<0.001$ treated vs control cells, One Way ANOVA analysis with Dunnett's multiple comparisons.
