# Supplementary material for: The TLR4-Active Morphine Metabolite Morphine-3-Glucuronide Does Not Elicit Macrophage Classical Activation In Vitro
Source: Front Pharmacol. 2016 Nov 17;7:441. doi: 10.3389/fphar.2016.00441 (PMC5112272; doi:10.3389/fphar.2016.00441)
Supplement: Supplementary file 6 [file Image_6.PDF]

Supplementary Figure 6

A

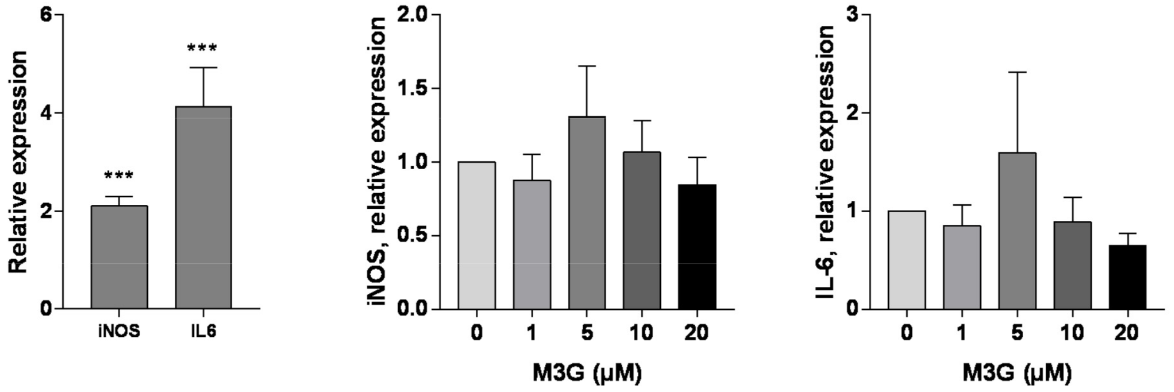

B

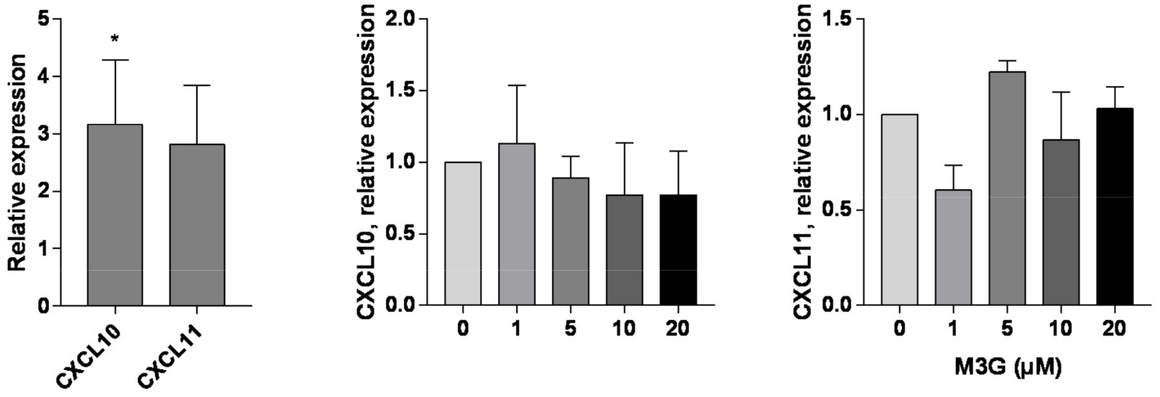

Supplementary figure 6: M3G does not alter TLR3-induced overexpression of M1 markers. A). RAW 264.7 cells were incubated with 10 ng/ml poly-ICLC, which induced a ~2 fold increase in iNOS and a ~4 fold increase in IL-6 mRNA expression. Results are shown as mean ± SEM, n=5 independent experiments. ***,$p<0.001$ treated vs control cells, one tailed Student's t test. When the cells were exposed to 10 ng/ml poly-ICLC together with 1-20 μM M3G, no decrease in gene induction was observed (One Way ANOVA analysis with Dunnett's multiple comparisons). Results re shown as mean ± SEM, n=3 independent experiments. **B)** THP-1 cells were incubated with 50 nM PMA for 48h and then exposed to 20 ng/ml poly-ICLC, which induced a ~3 fold increase in CXCL10 and CXCL11 mRNA expression. Results are shown as mean ± SEM, n=6 independent experiments. *,$p<0.05$ treated vs control cells, one tailed Student's t test. When THP1 cells were exposed to 20 ng/ml poly-ICLC together with 1-20 μM M3G, no decrease in gene induction was observed (One Way ANOVA analysis with Dunnett's multiple comparisons). Results re shown as mean ± SEM, n=3 independent experiments
